# Supplementary figures and images for: When did you leave home? Reconstructing juvenile life histories of coastal Washington adult coho salmon (Oncorhynchus kisutch) using otolith structure and chemistry
Source: PLoS One. 2026 Jan 13;21(1):e0340362. doi: 10.1371/journal.pone.0340362 (PMC12798972; doi:10.1371/journal.pone.0340362)

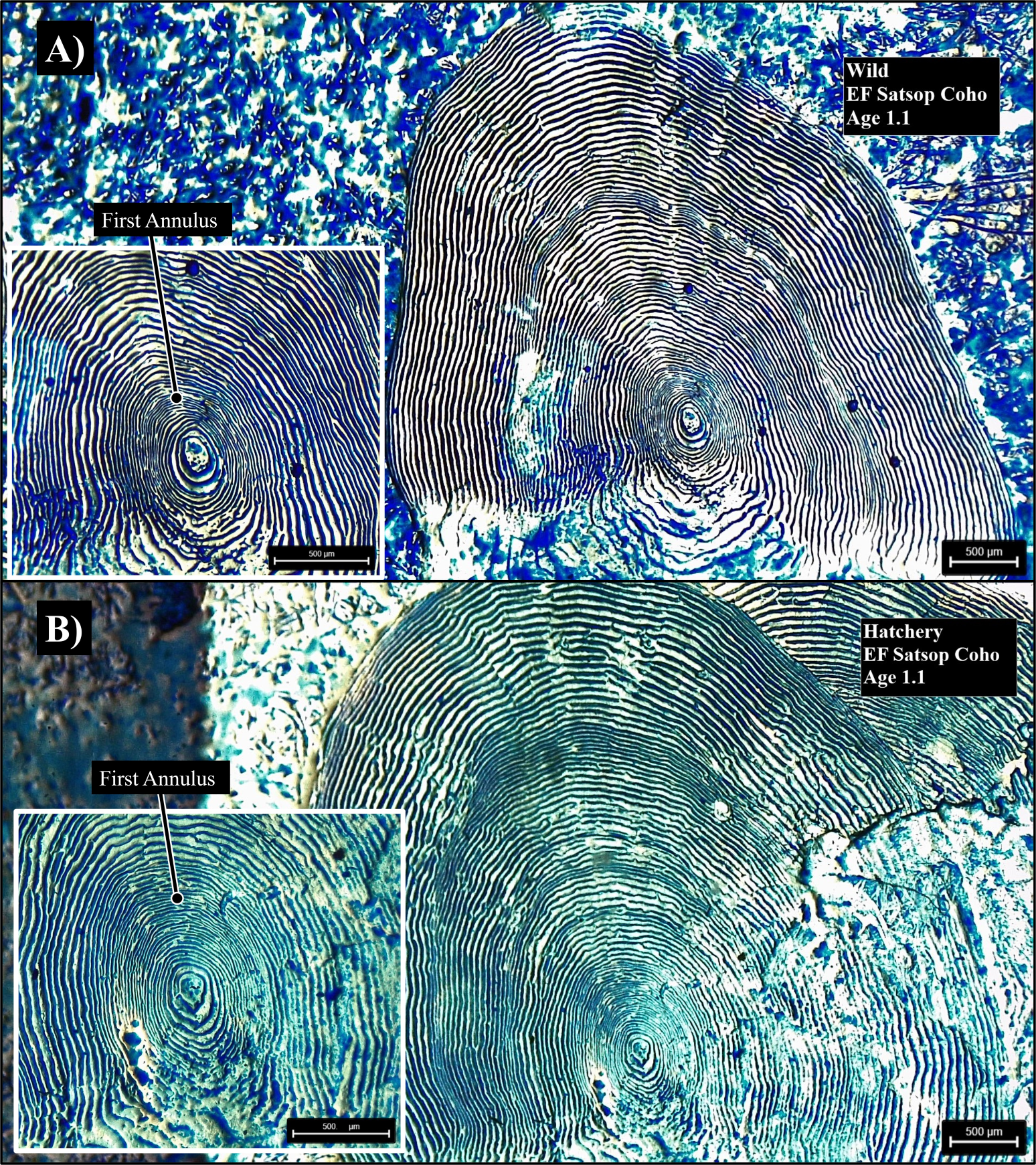

Supplement: S1 Fig — We used the size and pattern of the first annulus and freshwater zone to determine origin type. Scales from wild fish have a smaller and more defined first annulus than hatchery fish. Hatchery fish also consistently contain freshwater growth checks. (TIF) [file pone.0340362.s001.tif]

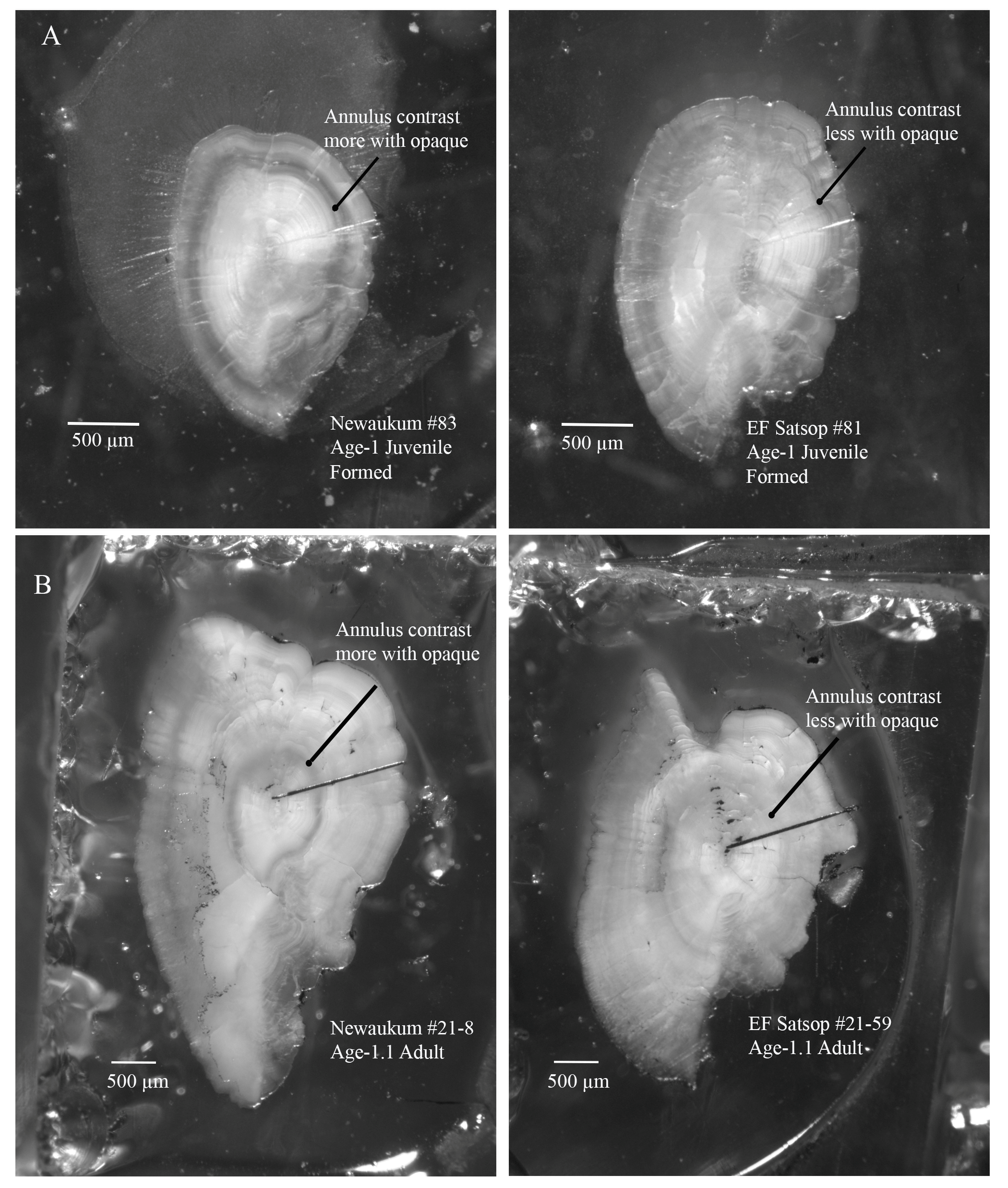

Supplement: S3 Fig — (TIF) [file pone.0340362.s003.tif]

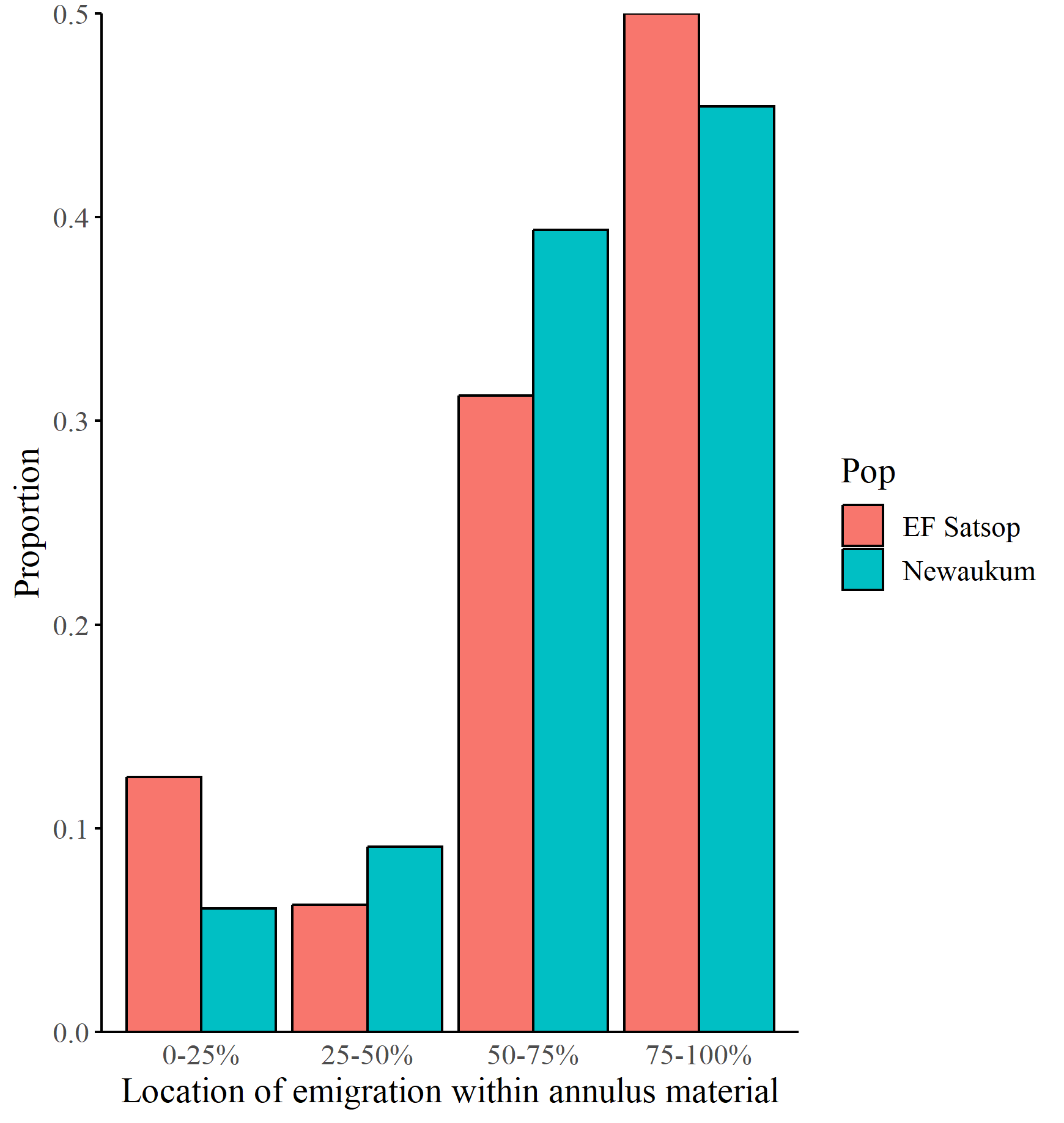

Supplement: S5 Fig — The 0th percentile indicates the beginning and the 100th percentile indicates the end of the annulus. (TIFF) [file pone.0340362.s005.tiff]
