# Supplementary material for: When did you leave home? Reconstructing juvenile life histories of coastal Washington adult coho salmon (Oncorhynchus kisutch) using otolith structure and chemistry
Source: PLoS One. 2026 Jan 13;21(1):e0340362. doi: 10.1371/journal.pone.0340362 (PMC12798972; doi:10.1371/journal.pone.0340362)
Supplement: Attachment — Submitted filename: Review for Anderson et al. PLOSONE.docx [file pone.0340362.s006.docx]

Major comments:

There are a few sources of uncertainty that could benefit from being mentioned in the discussion. If annuli formation is related to the thermal condition of the stream, it possible that timing of annuli formation may vary from year to year. This is unlikely to dramatically change your results, but it could be mentioned as a consideration in the discussion, especially if this approach is to be conducted over several brood years or in places where the thermal regime could differ dramatically among years. An additional point to mention in the discussion is the inherent survivability bias from adult otolith studies. The proportional life history may hold if it is assumed that all life history types experience similar mortality, however, they may also be the result of variable survivability among these LH types.

Minor Comments :

L52-53: The description here could be clarified. This effect does not necessarily mean population abundance remains consistent but rather that variability in population level productivity is buffered compared to that of the components.

L 91-93: This sentence should be reworded for clarity. The key point is that Strontium values (in relation to Calcium) vary between FW and Marine environments (but also among fw environments) and are reflected in the otolith. However, there are some freshwater environments with high strontium.

L95: identifying is more accurately worded as “measuring”, “counting”, or “analyzing”

L 93-96: It may be helpful to mention that otolith growth is not only related to overall growth but is directly proportional in most cases. This will further support the back-calculation in the rest of the paper.

L509: This citation does not seem to be correctly formatted. I am assuming you’re referencing Brennan et al., 2019 ; Shifting habitat mosaics and fish production across river basins. Please check this and other citations which may not be fully formatted correctly.
