# Supplementary material for: When did you leave home? Reconstructing juvenile life histories of coastal Washington adult coho salmon (Oncorhynchus kisutch) using otolith structure and chemistry
Source: PLoS One. 2026 Jan 13;21(1):e0340362. doi: 10.1371/journal.pone.0340362 (PMC12798972; doi:10.1371/journal.pone.0340362)
Supplement: Attachment — Submitted filename: Response to Reviewers.docx [file pone.0340362.s008.docx]

Dear Editor,

Please find our responses to each of the reviewers’ specific comments below. To address the six journal requirement items:

1- File formatting updated to meet PLOS requirements.
2- We have removed the mention of funding from the acknowledgements and no longer have any mention of funding in the manuscript.
3- Data availability has not changed. Data will go live on data repository if/when manuscript is accepted as indicated in our original data availability statement.
4- Data used for creating the map comes from 2 public domain USGS datasets that are now included in the references - National Hydrography Dataset (NHD) and Watershed Boundary Dataset (WBD). There is no copyrighted material in figure 1.
5- N/A
6- We’ve edited a few citations to meet PLOS citation formatting. We’ve also added a few citations and have indicated these in the response to reviewers below. All of this is captured in our updated manuscript document with tracked changes.

**Reviewer #1**:

The research seeks to identify life history types of coho salmon in two tributaries to the Chehalis river in Washington state using the chronological properties of otoliths alongside chemical signatures indicative of marine entry or estuary use. Through these methods, the authors identify several life history types not accounted for in current fisheries management, suggesting additional life history types which may be contributing to a portfolio effect and uncertainty in run size estimates. The work is robust and considers several potential scenarios which may cause uncertainties in their own data. There is some room to add mention of additional uncertainties, however, the results presented here appear to be well supported and suggest a framework for similar monitoring of life history diversity using otolith chemical analysis. Specific suggestions and comments are uploaded as an attachment.

Major comments:

There are a few sources of uncertainty that could benefit from being mentioned in the discussion. If annuli formation is related to the thermal condition of the stream, it possible that timing of annuli formation may vary from year to year. This is unlikely to dramatically change your results, but it could be mentioned as a consideration in the discussion, especially if this approach is to be conducted over several brood years or in places where the thermal regime could differ dramatically among years. An additional point to mention in the discussion is the inherent survivability bias from adult otolith studies. The proportional life history may hold if it is assumed that all life history types experience similar mortality, however, they may also be the result of variable survivability among these LH types.

*Annual temp variation – We agree that this could influence the timing of annulus formation between years. We have added a paragraph near the end of the discussion addressing this point.*

*Survival bias – We are aware of survivability concerns that arise when using adult (survivor) samples in studies to draw conclusions, particularly about juvenile components of populations. However, in this study we were specifically interested in which life history types are surviving to adulthood to reproduce. We are not making assumptions about how these adult proportions translate to the proportions of juveniles outmigrating in the different groups. In the introduction and the discussion, we bring up the likely survival differences between fish that follow different life history strategies and discuss the implications/issues this raises with current management practices that don’t take this into account.*

Minor Comments:

L52-53: The description here could be clarified. This effect does not necessarily mean population abundance remains consistent but rather that variability in population level productivity is buffered compared to that of the components.

*We have adjusted this sentence to improve clarity and address this concern.*

L 91-93: This sentence should be reworded for clarity. The key point is that Strontium values (in relation to Calcium) vary between FW and Marine environments (but also among fw environments) and are reflected in the otolith. However, there are some freshwater environments with high strontium.

*We’ve rewritten this paragraph to add more basic otolith and microchemistry background info to give PLOS’s readers more context including referencing the Sr to Ca ratio (“Sr:Ca”) as opposed to just Sr itself. We believe these changes have addressed this concern and improved clarity of the entire section.*

L95: identifying is more accurately worded as “measuring”, “counting”, or “analyzing”

*Change made, now use “counting.”*

L 93-96: It may be helpful to mention that otolith growth is not only related to overall growth but is directly proportional in most cases. This will further support the back-calculation in the rest of the paper.

*We have added some additional basic otolith information to this section to give PLOS’s broad audience some context. This includes indicating that otolith-body length growth relationship is directly related.*

L509: This citation does not seem to be correctly formatted. I am assuming you’re referencing Brennan et al., 2019 ; Shifting habitat mosaics and fish production across river basins. Please check this and other citations which may not be fully formatted correctly.

*We have corrected this citation and a few others in the references section, thanks for catching that.*

**Reviewer #2**

General comments:
This manuscript reports important research that offers substantial insights for management and decision-making. While mostly well written there are several instances where the writing is overly vague, especially in the methods section. A light revision with a focus on improving the precision of language would strengthen the manuscript.

Because PLOS ONE is a general-purpose journal with a wide readership, the authors should not assume that all readers are familiar with otoliths. A short paragraph in the introduction explaining what otoliths are and how they grow would improve accessibility for a broader audience.

*We agree with this recommendation and have added a background paragraph to the Introduction with basic otolith and microchemistry information to give context for PLOS’s audience that may not be familiar with otoliths.*

Research measuring Sr/Ca in otoliths often includes Ba/Ca to help differentiate estuarine from marine water. I am curious why barium was not measured here as it might have provided insight into the mystery surrounding the second and third annuli having a freshwater appearance. A quadrupole ICPMS should allow Sr, Ba and Ca to be analyzed simultaneously.

*The ICPMS also measured Ba for these samples. Unlike Sr, Ba can change drastically with temperature (as well as salinity, metabolism, etc.) which can confound interpretations. Miller et al. 2011 (cited in manuscript) shows these challenges with using Ba. We did not end up using the Ba information for our life history distinctions, timing of marine entry identification, or any of our analyses, so we did not include Ba in the manuscript.*

Line by line comments:
Line 28: “…timing of otolith growth…” It is unclear what you are referring to here.

*Change made to improve clarity. Now reads “First, we established the timing of otolith annulus formation…”*

Line 31: The term “annulus measurements” is unclear. What did you measure on the annuli? Distance? Count?’

*Change made to improve clarity. Now reads “We then used otolith microchemistry in conjunction with measurements of freshwater annulus location along the microchemistry transect from returning adults (n=173) to describe the timing of freshwater emigration of fish from these two tributaries.”*

Lines 93 and 96: Revise the sentence beginning at line 93 so that its first half stands as an independent sentence. Then combine the latter half of that sentence with the sentence beginning at line 96 to avoid repetition and improve the overall flow.

*We have rewritten this otolith background section of the introduction including this sentence.*

Line 138: Was this a carcass survey? Was it by boat, snorkel or foot?

*Clarification added to this section. They were a combination of foot and float carcass surveys.*

Line 164: How are you delineating the quadrants?

*Quadrants were determined visually based on otolith morphology. Specifically using the rostrum and dorsal edge as landmarks.*

Line 174: This paragraph can use clarification to better communicate your process. When discussing the aging of juvenile otoliths readers might think you are performing daily increment reads so specify that these are annual reads and you are differentiating age-0 from spring age-1. Portions of the methods that were used on both juveniles and adult otoliths should be independent of the paragraph dedicated to juvenile methods.

*We agree with this suggestion. We have separated the ageing and annulus development sections into their own paragraphs. We have clarified that juvenile ages are annual ages to avoid any daily increment confusion. We have also clarified that ageing occurred for both juvenile and adult otoliths and described the process/notation for each.*

Line 177: Please specify which otolith structures you are referring to. How is assigned age different than [date of capture]-[Jan 1st]?

*We have rewritten and clarified this section as per the previous line item response.*

Line 183: How did the readers resolve disagreements? How many samples were resolved in this way?

*We’ve edited this sentence to clarify how disagreements were resolved. We did not record how many disagreements there were, but it was a minority. Very few age disagreements (maybe 2?) and a handful of development type disagreements.*

Line 191: Change “corresponding Sr:Ca” to “corresponding Sr:Ca profile” or “corresponding Sr:Ca life history profile.”

*Change made to “Sr:Ca profile”*

Line 196: Expand on this sentence. You don’t need to define each individual life history type here but more detail than “A unique combination” would be helpful.

*We have added some additional description to help clarify how we defined and classified the different life history types.*

Line 214: Did you investigate other models for fork length back-calculation other a linear regression?

*We did not investigate other back calculation methods for this study. Two of the co-authors of this manuscript have published another study comparing back calculation methods (linear and proportional) for chinook. They found direct (linear) back-calculation models to be accurate and more robust than the proportional method because the proportional method cannot be applied to adults without violating its assumptions. The proportional assumption is that otolith size–fish size relationship is relatively consistent, which is not true for adults whose relationship changes between the freshwater and marine environment. We developed the best linear model we could by using fish from the study populations via our juvenile sampling.*

*Claiborne AM, Campbell LA. Evaluation of back‐calculated size and timing estimates for juvenile Chinook Salmon using otolith structure and chemistry. Transactions of the American Fisheries Society. 2016 May 1;145(3):493-501.*

*Miller JA, Gray A, Merz J. Quantifying the contribution of juvenile migratory phenotypes in a population of Chinook salmon Oncorhynchus tshawytscha. Mar Ecol Prog Ser. 2010;408:227–40.*

Line 237: Just R? Were any packages used?

*Citations for packages have been added.*

Line 255-256: Rewrite the two sentences in these lines into one.

*Change made.*

Line 271: Observing the relative contrast of the annuli between the two sites comes off as rather subjective. Image Pro has the functionality to measure luminosity to objectively compare contrast between the two study areas.

*Thanks for the suggestion, we didn’t think to use that Image Pro function. This observed difference was only intended as a qualitative observation and we don’t feel it necessary to quantify the contrast difference for the current study.*

Line 300: Did you explain somewhere the difference between freshwater and marine annuli and is there a citation?

*I’ve expanded on the description of why this presented like a freshwater annulus in this section. Unfortunately, we do not have a good reference for this distinction.*

Line 309: Change “Sr:Ca” to “Sr:Ca profiles” or “Sr:Ca life history profiles”

*Change made.*

Line 425: Add the word “is” between adjustment and needed

*Change made.*
